# Brazilian transcultural adaptation of an instrument on physicians' knowledge and attitudes towards dementia

**DOI:** 10.1590/1980-57642015DN93000006

**Published:** 2015

**Authors:** Alessandro Ferrari Jacinto, Erika Correa de Oliveira, Vanessa de Albuquerque Citero

**Affiliations:** 1 M.D., Ph.D. Professor Doutor Assistente, Disciplina de Geriatria (Departamento de Clínica Médica), Faculdade de Medicina de Botucatu, Universidade Estadual Paulista Júlio de Mesquita Filho / Pós-doutorando do Departamento de Psiquiatria da Escola Paulista de Medicina, Universidade Federal de São Paulo.; 2 Psychologist. Mestranda do Departamento de Psiquiatria, Escola Paulista de Medicina, Universidade Federal de São Paulo.; 3 M.D., Ph.D. Professora do Programa de Pós- Graduação do Departamento de Psiquiatria, Escola Paulista de Medicina, Universidade Federal de São Paulo.

**Keywords:** dementia, aged, knowledge, physicians

## Abstract

**Objective:**

The aim of this study was to obtain a Brazilian transcultural adaptation of an instrument developed in the United Kingdom for assessing the knowledge and attitudes towards dementia by physicians.

**Methods:**

The "Knowledge Quiz" (KQ) contains 14 items on epidemiology, diagnosis and management of dementia, while the "Attitude Quiz" contains 10 sentences about physicians' thoughts on the management of demented patients. The Quizzes were translated, back-translated and the resultant version applied to five physicians.

**Results:**

The transcultural equivalence process was performed and four items of the KQ needed adapting to the Brazilian context. After changes suggested by a panel of specialists, the final version was applied to another five physicians and the transcultural equivalence considered adequate.

**Conclusion:**

The Brazilian version of the instrument was successfully transculturally adapted for future validation and application in Brazil.

## Abstract (pt)

**Objetivo:**

Este estudo objetivou obter a adaptação transcultural de um instrumento desenvolvido no Reino Unido para avaliar o conhecimento e as atitudes dos médicos brasileiros diante de pacientes com demência.

**Métodos:**

O "Questionário de Conhecimento" (QC) tem 14 itens sobre epidemiologia, diagnóstico e tratamento da demência; e o "Questionário de Atitudes" tem 10 sentenças sobre os pensamentos dos médicos no cuidado de pacientes com demência. Eles foram traduzidos, retrotraduzidos e uma versão foi aplicada para cinco médicos.

**Resultados:**

A equivalência transcultural foi realizada, e quatro itens do QC precisaram ser adaptados para o contexto brasileiro. Após as mudanças sugeridas por um comitê de especialistas, a versão final foi aplicada para outros cinco médicos e a equivalência transcultural foi considerada adequada.

**Conclusão:**

A versão brasileira do instrumento foi adaptada transculturalmente com sucesso e está pronta para ser validada e aplicada no Brasil.

## INTRODUCTION

TThe prevalence of dementia in Brazil has increased in the last three decades amidst a rapidly aging population.^1^ An estimated 24.3 million people worldwide have dementia with an incidence of 4.6 million new cases each year. According to the same group, cases of dementia are set to double every 20 years, rising to 81.1 million by 2040. Currently, 61% of all cases of dementia are found in underdeveloped countries.^2^

Physicians often overlook cognitive impairment (CI) in the elderly, especially in its early stages.^3-6^ umber of countries. In Brazil, only one Brazilian study has investigate this issue.^7^ Considering evidence-based medicine, it is unclear whether early detection of dementia improves patients' outcomes, although cognitive impairment assessment is part of the global geriatric assessment.^8,9^

Few studies^10-13^ have identified dementia knowledge deficits among health workers and caregivers. Basically, two instruments designed for this purpose have been used in these studies: the Alzheimer's Disease Knowledge Scale^14^ and the "Knowledge Quiz and Attitude Quiz".^10^ It is important for this type of instrument to be short and easy to answer. Instruments should also have good reliability in measuring what they propose to assess. International use of the instrument is also of importance since it enables comparisons between different medical educational systems. Of the two instruments cited, the "Knowledge Quiz and the Attitude Quiz" by Turner et al.^10^ seems a promising tool for the assessment of CI by Brazilian General Practitioners, having been used for this purpose in the United Kingdom.

The aim of this study was to obtain a cross-cultural adaptation of an instrument^10^ previously used in the United Kingdom for assessing the knowledge and the attitudes towards dementia of Brazilian physicians.

## METHODS

The translation of the instrument comprising the Knowledge Quiz (KQ) and Attitude Quiz (AQ) questionnaires was authorized by their authors and this project was approved by the Ethics Committee of the *Escola Paulista de Medicina, Universidade Federal de São Paulo*.

**Subjects.** Numerous cross-cultural adaptation studies have shown that the instrument's final version can be applied to a small sample of subjects since its characteristics accommodate diversity of individuals.^15^ In this study, the participants of the cross-cultural adaptation of the KQ and AQ were five recently graduated physicians from different Brazilian medical schools. Five other physician could be assessed if necessary, in a second application of the instrument. The inclusion criterion was to be on the first year of a medical residence program of the "Escola Paulista de Medicina, Universidade Federal de São Paulo". There were no exclusion criteria.

The physicians were invited to participate in the study during a "Welcome section" of the residence programs run by the institution. The physicians were randomly selected and asked to fill out the instrument. None of the physicians refused to participate.

**Instruments.** The Knowledge Quiz (Table 1, original) consists of 14 tests with five multiple choice answers, including "I don't know". Only one of the answers is correct. This questionnaire covers three issues on dementia: epidemiology, diagnosis and management. The Attitude Quiz (Table 2, original) contains 10 sentences about physicians' thoughts on the management of demented patients on a 5-point "Likert type" scale (from "strongly agree" to "strongly disagree").

**Table 1:** The Knowledge Quiz about dementia in its United Kingdom original version10 and Brazilian cross-cultural adapted version.

Question	United Kingdom original version*	Brazilian cross-cultural adapted version**
1	A general practitioner with a list of 1,500–2,000 people can expect to have the following number of people with dementia on their list: A. 1–6 B. 7–11 C. 12–20 D. 21 or more E. I don’t know	*Um clínico geral com uma lista de 1000 pessoas com 60 anos ou mais deve esperar ter o seguinte número aproximado de pessoas com demência nesta lista: * *A. 10 * *B. 500 * *C. 200 * *D. 70 E. Não sei*
2	By 2021, the prevalence of dementia in the general population in the UK is expected to: A. Decrease slightly B. Remain approximately the same C. Increase slightly D. Nearly double E. I don’t know	*A partir dos 65 anos de idade, a prevalência de demências: * *A. Dobra a cada 5 anos * *B. Dobra a cada 10 anos * *C. Dobra a cada 15 anos * *D. Dobra a cada 20 anos E. Não sei*
3	One of the risk factors for the development of Alzheimer’s disease is: A. Hardening of arteries B. Age C. Nutritional deficiencies D. Exposure to aluminium E. I don’t know	*Um dos fatores de risco para o desenvolvimento da doença de Alzheimer é: * *A. Endurecimento das artérias * *B. Idade * *C. Deficiências nutricionais * *D. Exposição ao alumínio E. Não sei*
4	All of the following are potentially treatable etiologies of dementia except: A. Hypothyroidism B. Normal pressure hydrocephalus C. Creutzfeldt–Jacob disease D. Vitamin B12 deficiency E. I don’t know	*Todas as seguintes são etiologias potencialmente tratáveis de demência, exceto: * *A. Hipotireoidismo * *B. Hidrocefalia de pressão normal * *C. Doença de Creutzfeldt-Jakob * *D. Deficiência de vitamina B12 E. Não sei*
5	A patient suspected of having dementia should be evaluated as soon as possible as: A. Prompt treatment of dementia may prevent worsening of symptoms B. Prompt treatment of dementia may reverse symptoms C. It is important to rule out and treat reversible disorders D. It is best to institutionalise a dementia patient early in the course of the disease E. I don’t know	*Um paciente com suspeita de demência deve ser avaliado assim que possível, pois: * *A. O tratamento imediato contra a demência pode impedir o agravamento dos sintomas * *B. O tratamento imediato contra a demência pode reverter os sintomas * *C. É importante descartar e tratar distúrbios reversíveis * *D. É melhor institucionalizar um paciente com demência já no início da doença E. Não sei*
6	Which of the following procedures is required to definitely confirm that symptoms are due to dementia? A. Mini-Mental State Exam B. Post mortem C. CAT scan of the brain D. Blood test E. I don’t know	*Qual dos procedimentos seguintes é necessário para confirmar definitivamente que os sintomas são causados pela demência? * *A. Mini Exame de Estado Mental * *B. Exame post mortem * *C. Tomografia do cérebro * *D. Exame de sangue E. Não sei*
7	Which of the following is not a necessary part of the initial evaluation of a patient with possible dementia? A. Thyroid function test B. Serum electrolytes C. Vitamin B and foliate levels D. Protein electrophoresis E. I don’t know	*Qual das alternativas não é uma parte necessária da avaliação inicial de um paciente com suspeita de demência? * *A. Exame de função da tireóide * *B. Eletrólitos séricos * *C. Níveis de vitamina B e ácido fólico * *D. Eletroforese de proteínas E. Não sei*
8	Which of the following sometimes resembles dementia? A. Depression B. Acute confusional state C. Stroke D. All of the above E. I don’t know	*Qual das alternativas pode se assemelhar à demência? * *A. Depressão * *B. Estado confusional agudo * *C. Derrame cerebral * *D. Todas os anteriores E. Não sei*
9	When a patient develops a sudden onset of confusion, disorienta­tion, and inability to sustain attention, this presentation is most consistent with the diagnosis of: A. Alzheimer’s disease B. Acute confusional state C. Major depression D. Vascular dementia E. I don’t know	*Quando um paciente apresenta um súbito inicio de confusão, desorientação e incapacidade de manter a atenção, esse quadro é mais compatível com o diagnóstico de: * *A. Doença de Alzheimer * *B. Estado confusional agudo * *C. Depressão maior * *D. Demência vascular E. Não sei*
10	Which of the following is nearly always present in dementia? A. Loss of memory B. Loss of memory and incontinence C. Loss of memory, incontinence and hallucinations D. None of the above E. I don’t know	*Qual das seguintes opções está quase sempre presente na demência? * *A. Perda de memória * *B. Perda de memória e incontinência * *C. Perda de memória, incontinência e alucinações * *D. Nenhuma das anteriores E. Não sei*
11	Which of the following clinical findings best differentiates vascular dementia from Azheimer’s? A. Word-finding problems B. Short-term (2-minute span) visual memory loss C. Stepwise disease course D. Presence of depression E. I don’t know	*Qual dos seguintes achados clínicos melhor diferencia a demência vascular da demência da doença de Azheimer? * *A. Problemas para encontrar palavras * *B. Perda de memória visual imediata (2 minutos) * *C. Desenvolvimento da doença em escada (patamares com estabilização, intercalados com declínio súbito) * *D. Presença de depressão E. Não sei*
12	The effect of anti-dementia drugs is to: A. Temporarily halt the disease in all cases B. Temporarily halt the disease in some cases C. Temporarily halt the disease in some cases but often causing liver damage D. Permanently halt the disease in some cases E. I don’t know	*O efeito dos medicamentos anti demência atua em: * *A. Interromper temporariamente a doença em todos os casos * *B. Interromper temporariamente a doença em alguns casos * *C. Interromper temporariamente a doença em alguns casos, mas freqüentemente causa danos ao fígado * *D. Interromper definitivamente a doença em alguns casos E. Não sei*
13	Which statement is true concerning the treatment of dementia patients who are depressed? A. It is usually useless to treat them for depression because feelings of sadness and inadequacy are part of the disease B. Treatments of depression may be effective in alleviating depressive symptoms C. Anti-depressant medication should not be prescribed D. Proper medication may alleviate symptoms of depression and prevent further intellectual decline E. I don’t know	*Qual afirmação é verdadeira sobre o tratamento de pacientes com demência que estão deprimidos? * *A. Geralmente é inútil tratá–los para a depressão, pois os sentimentos de tristeza e inadequação são parte da doença * *B. Tratamentos contra a depressão podem ser eficazes no alívio dos sintomas depressivos * *C. Medicamentos antidepressivos não devem ser prescrevidos * *D. A medicação correta pode aliviar os sintomas da depressão e prevenir um futuro declínio intelectual E. Não sei*
14	Which of the following best describes the functions of the Alzheimer’s Disease Society? A. Central research, information and campaigning role B. Provision of local support and education to carers C. Providing day and home care for dementia patients D. All of the above E. I don’t know	*A ABRAZ é a associação brasileira que fornece informações para pacientes e cuidadores com qual propósito? * *A. Ajudar as pessoas a entenderem melhor a doença, para que possam lidar de maneira mais adequada com os sintomas e tratamentos * *B. Atendimento médico ambulatorial gratuito * *C. Captação de pessoas com demência para pesquisas * *D. Todas as anteriores E. Não sei*

* Correct answers in original questionnaire: 1 C; 2 C; 3 B; 4 C; 5 C; 6 B; 7 D; 8 A; 9 D; 10 B; 11 C; 12 B; 13 B; 14 D.

** Correct answers in Brazilian version: 1 D; 2 A; 3 B; 4 C; 5 C; 6 B; 7 D; 8 A; 9 D; 10 B; 11 C; 12 B; 13 B; 14 A.

**Table 2:** “Attitude Quiz” of physicians toward dementia.

Item	United Kingdom original version	Brazilian cross-culturally adapted version
1	Much can be done to improve the quality of life of carers of people with dementia	*Muito pode ser feito para melhorar a qualidade de vida de cuidadores de pessoas com demência*
2	Families would rather be told about their relative’s dementia as soon as possible	*As famílias preferem ser informadas a respeito da demência de seu parente o mais rápido possível*
3	Much can be done to improve the quality of life of people with dementia	*Muito pode ser feito para melhorar a qualidade de vida das pessoas com demência*
4	Providing diagnosis is usually more helpful than harmful	*Fornecer diagnóstico geralmente é mais útil do que prejudicial*
5	Dementia is best diagnosed by specialist services	*A demência é mais bem diagnosticada em serviços especializados*
6	Patients with dementia can be a drain on resources with little positive outcome	*Os pacientes com demência podem esgotar recursos com resultado pouco positivo*
7	It is better to talk to the patient in euphemistic terms	*É melhor conversar com o paciente utilizando eufemismos*
8	Managing dementia is more often frustrating than rewarding	*Tratar a demência costuma ser mais frustrante do que gratificante*
9	There is little point in referring families to services as they do not want to use them	*Não vale a pena direcionar as famílias para serviços especializados quando elas não querem usá-los*
10	The primary care team has a very limited role to play in the care of people with dementia	*A equipe de atenção primária tem um papel muito limitado no cuidado de pessoas com demência*

* Scores in original questionnaire: (1) strongly agree, (2) agree, (3) neither, (4) disagree, (5) strongly disagree.

** Scores in Brazilian version: (1) *concordo plenamente*, (2) *concordo*, (3) *não concordo nem discordo*, (4) *discordo*, (5) *discordo plenamente*.

**Procedures.** The following cross-cultural adaptation method was applied:^16^

The quiz items were translated from English into Portuguese by two bilingual professionals who were independent and were aware of the aims of the study. The two versions were compared by the authors, a consensual translation was created and the two bilingual professionals subsequently approved it.

The consensual translation was back-translated into English by two other bilingual professionals. These two versions were compared and a consensual back-translated instrument created to guarantee semantic equivalence of the items. The two bilingual professionals subsequently approved it.

Finally, cross-cultural equivalence was performed to identify those items that were not readily understood. To this end, the answer option "not applied" was included for each item and the quizzes applied to five physicians. The AQ raised no doubts among these physicians and the consensual translated version was established as the final Brazilian version (Table 2). However, four items of the KQ needed adapting (questions 1, 2, 11 and 14). A panel of specialists (a geriatrician and a psychiatrist, both specialized in cognitive impairment in elderly) suggested changes of an idiomatic, cultural and conceptual nature:

Questions 1 and 2 describe dementia prevalence in the general population in an unusual way for Brazilian medical culture, and were therefore modified:

(i) Question 1 , from "a general practitioner with a list of 1,500–2,000 people can expect to have the following number of people with dementia on their list" to "a general practitioner with a list of 1,000 people aged 60 years or older can expect to have the following number of people with dementia on their list";
(ii) Question 2, from "by 2021, the prevalence of dementia in the general population in the UK is expected to" to "from 65 years of age, the prevalence of dementia...".

An explanation was included (in brackets) about the meaning of "stepwise disease course" on answer "c" of question 11.

On question 14, Alzheimer's Disease Society was changed to Brazilian Alzheimer's Disease Association.

After these changes, the KQ was reapplied to another five physicians and the cross-cultural equivalence was performed with no doubts raised. The definitive Brazilian version of the KQ is given in Table 1.

Descriptive analyses of the subjects in both applications were performed.

## RESULTS

Table 1 shows the KQ and Table 2 depicts the AQ instrument, in both the original and cross-culturally translated versions.

Table 3 contains the distribution of socio-demographic and graduation characteristics of both applications. In the first application, the residents were 40% female, with mean age of 26.2 (±1.9) years, and mean time since graduation of 16.4 (±13.1) months; 80% were on a specialization program of internal medicine, and 80% graduated from a Federal medical school. Sixty percent of the physicians considered they had good training on the cognitive impairment issue during medical school. In second application, the residents were also 40% female, with mean age of 27.0 (±2.6) years, and mean time since graduation of 11.6 (±13.1) months; 60% were on a specialization program of internal medicine, and 80% graduated from a private medical school. Sixty percent of the physicians also considered they had good training on the cognitive impairment issue during medical school.

**Table 3:** Distribution of demographic and schooling characteristics of physicians who answered the instrument applications.

Application	Gender	Age (years)	Time since graduation (months)	Which medical school did you graduate from?	Medical residency field	Did you have any dementia training during medical school?
1^st^	Female	27	26	Universidade Federal de São Paulo	Internal Medicine	Yes
1^st^	Male	25	26	Universidade Federal do Piauí	Internal Medicine	No
1^st^	Male	24	2	Faculdade de Ciências Médicas da Santa Casa de São Paulo	Internal Medicine	Yes
1^st^	Female	26	2	Universidade Federal de São Paulo	Internal Medicine	Yes
1^st^	Male	29	26	Universidade Federal da Paraíba	Surgery	No
2^nd^	Male	24	2	Universidade de Marilia	Internal Medicine	Yes
2^nd^	Male	26	2	Faculdade de Medicina do ABC	Internal Medicine	Yes
2^nd^	Female	31	2	Centro Universitário Lusiadas	Internal Medicine	No
2^nd^	Female	27	26	Universidade do Grande Rio	Surgery	Yes
2^nd^	Male	27	26	Universidade Federal de Rondônia	Surgery	No

## DISCUSSION

In terms of public health, physicians' knowledge about dementia in elderly and the attitude they have toward demented patients is important to guarantee reliable clinical care for patients with dementia. Dementia cases have been increasing in Brazil and worldwide^1,2^, representing an issue of concern across all spheres of Medicine. Straight-forward instruments that can better train physicians to deal with dementia are fundamental. The internationality of these instruments is also important to allow comparisons of medical education, thus cross-cultural adaptation is a method of promoting this exchange of experience.

There were no apparent difficulties translating and back-translating the instrument, but the conceptual and semantic equivalence raised more doubts for the KQ than the AQ. Ultimately, both questionnaires were successfully cross-culturally adapted, and the Brazilian version of the KQ about dementia and the AQ toward dementia patients can be used to compare Brazilian physicians to other physicians worldwide. The panel of specialists contributed to the cultural equivalence of KQ by changing some questions about epidemiology. Brazilian studies on prevalence express these numbers as percentages according to age.^17^ The importance of dementia as an epidemic issue in public health was the priority to be emphasized in questions 1 and 2. These questions address basic information about dementia prevalence that all physicians in Brazil should know. Question 14 also underwent adaptation to the Brazilian context, reflecting the role of a Brazilian equivalent of the Alzheimer Disease Society. On the other hand, the change in question 11 was more straight-forward, entailing adaptation to include a linguistic explanation of the term "stepwise disease course".

A limitation of this study was the profile of the physicians to whom the Brazilian version was applied. These physicians were young and recently graduated which may have facilitated comprehension of the instrument, as compared to older doctors. Nevertheless, these recently graduated physicians represent the next generation of physicians to attend as general practitioners in public health.

In the dementia field, primary care physicians in the United Kingdom have been studied periodically by the KQ and AQ instrument. The authors of the present study intend to discuss the quality of teaching on this issue in Brazil.

In conclusion, the Brazilian version of the instrument was successfully cross-culturally adapted for future validation and application in Brazil, serving to improve health-care assistance in primary care.

## References

[1] Rizzi L, Rosset I, Roriz-Cruz M (2014). Global epidemiology of dementia: Alzheimer's and vascular
types. Biomed Res Int.

[2] Ferri CP, Prince M, Brayne C (2005). Global prevalence of dementia: a Delphi consensus
study. Lancet.

[3] (2006). Neurological Disorders: public health challenges.

[4] Gifford DR, Cummings JL (1999). Rating dementia screening tests: methodologic standards to rate
their performance. Neurology.

[5] Valcour VG, Masaki H, Curb JD, Blanchette PL (2000). The detection of dementia in the primary care
setting. Arch Int Med.

[6] Finkel SI (2003). Cognitive screening in the primary care setting: the role of
physicians at the first point entry. Geriatrics.

[7] Jacinto AF, Brucki SMD, Porto CS, Martins MA, Nitrini R (2011). Detection of cognitive impairment in the elderly by general
internists in Brazil. Clinics.

[8] Lin JS, O'Connor E, Rossom RC, Perdue LA, Eckstrom E (2013). Screening for cognitive impairment in older adults: a systematic
review for the US Preventive Services Task Force. Ann Int Med.

[9] Borson S, Frank L, Bayley PJ (2013). Improving dementia care: The role of screening and detection of
cognitive impairment. Alzheimers Dement.

[10] Turner S, Iliffe S, Downs M (2004). General practitioners' knowledge, confidence and attitudes in the
diagnosis and management of dementia. Age Ageing.

[11] Smyth W, Fielding E, Beattie E (2013). A survey-based study of knowledge of Alzheimer's disease among
health care staff. BMC Geriatr.

[12] Hughes J, Bagley H, Reilly S, Burns A, Challis D (2008). Care staff working with people with dementia: training, knowledge
and confidence. Dementia.

[13] Robinson A, Eccleston C, Annear M (2014). Who knows, who cares? Dementia knowledge among nurses, care
workers, and family members of people living with dementia. J Palliat Care.

[14] Carpenter B, Balsis S, Otilingam P, Hanson P, Gatz M (2009). The Alzheimer's Disease Knowledge Scale: Development and
psychometric properties. Gerontologist.

[15] Guillemin F, Bombardier C, Beaton D (1993). Cross-cultural adaptation of health-related quality of life
measures: literature review and proposed guidelines. J Clin Epidemiol.

[16] Canino GJ, Bravo M (1985). The Adaptation and Testing of Diagnostic and Outcome Measures for
Cross-Cultural Research. Int Rev Psychiatry.

[17] Nitrini R, Caramelli P, Herrera E (2004). Incidence of dementia in a community-dwelling Brazilian
population. Alzheimer Dis Assoc Disord.

